# A Case of Recurrent Meningitis Secondary to Inadequate Repair of Traumatic Orbital Fracture.

**DOI:** 10.51894/001c.144465

**Published:** 2025-09-30

**Authors:** Grant Goodfellow

**Affiliations:** 1 Department of Ophthalmology Henry Ford Warren

## 83

### INTRODUCTION

Risk of developing bacterial meningitis is increased in patients with abnormal cerebrospinal fluid (CSF) connections. Proper surgical repair following orbital trauma and enucleation is crucial to close pathways for direct entry of external microbes into the CSF. Herein, we present a patient with recurrent bacterial meningitis due to a remote history of a gunshot wound to the right orbit, resulting in bony defects and anomalous CSF connections.

### CASE DESCRIPTION

A 31-year-old female with a remote history of right-eye enucleation following gunshot injury presented to the emergency department after being found unresponsive at home. History revealed that she never received proper repair of her skull fractures and has had three subsequent episodes of meningitis. Initial examination revealed an erythematous right orbit with foul-smelling, purulent discharge, without a conformer or prosthesis in place. Labs revealed a neutrophil-predominant leukocytosis, and maxillofacial computed tomography (CT) suggested chronic deformities of the face related to her prior injury. A presumptive diagnosis of meningitis was made, blood and wound cultures were obtained, and empiric antimicrobial treatment was initiated. The patient’s mental status improved with treatment. A lumbar puncture (LP) was completed and CSF cultures collected. The CSF formula revealed a significant polymorphonuclear leukocyte-predominant pleocytosis. Following the LP, clear fluid was observed draining from the patient’s nares; she then endorsed a history of recurrent orbital leakage since her enucleation. Concern for CSF leak triggered a neurosurgical consultation. The decision was made to proceed with a skull-base surgical repair to properly close the anomalous CSF pathways, ideally preventing future episodes of recurrent meningitis.

### CONCLUSIONS

Skull base defects can present a pathway for direct entry of microorganisms into the CSF. Surgical repair of these bony defects with closure of anomalous CSF pathways is crucial for prevention of meningitis. Our patient’s history of orbital gunshot injury and inadequate fracture repair following enucleation supports the connection between recurrent meningitis in the setting of compromised orbital anatomy. This case also highlights the importance of obtaining cultures from both the presumed site of microorganism entry, as well as CSF studies and cultures in order to best direct treatment.

